# Treating Complex Regional Pain Syndrome Using Counterstrain: A Novel Approach

**DOI:** 10.7759/cureus.10948

**Published:** 2020-10-14

**Authors:** Karl Schranz, Daltrey Meitz, Bethany Powers, Adrienne Ables

**Affiliations:** 1 Osteopathic Manipulative Medicine, Edward Via College of Osteopathic Medicine, Spartanburg, USA

**Keywords:** crps, chronic pain, neuropathic, omt, case report, counterstrain

## Abstract

Complex regional pain syndrome (CRPS) is a challenging disease to treat and requires a multidisciplinary and multimodal approach. We discuss the use of a novel approach to counterstrain using irritants - as identified by the patient - to induce symptoms of neuropathy and paresthesia and treat these symptoms as if they were counterstrain tender points. This treatment approach to CRPS has not previously been described in osteopathic manipulative treatment (OMT) literature, including Foundations of Osteopathic Medicine.

A 23-year-old female presented with an array of symptoms consistent with complex regional pain syndrome in her right foot and lower leg that had been gradually worsening for approximately one year. She had been treated with physical therapy, medications, injections, orthotics, and a transcutaneous nerve stimulation (TENS) unit, all of which provided temporary symptomatic relief but had not treated the underlying disease. By utilizing the mentioned osteopathic approach to treat the neuropathic aspect of her CRPS, we were able to produce a lasting resolution of her symptoms and improve her loss in proprioception and temperature discrimination in the affected limb.

A counterstrain should be considered a reasonable option to assist in the treatment of complex regional pain syndrome. This new treatment approach does not require extensive training or experience with osteopathic manipulative treatment, nor does it take much time to administer. Thus, it could be easily learned and utilized by many standard practitioners for patients with complex regional pain syndrome. In addition, given its low intensity and passive approach, it more likely to be tolerated well by patients. Understanding the challenging nature of treating complex regional pain syndrome, this report aims to be helpful in adding to the general fund of knowledge regarding this condition and the possible treatments. We fully understand that the safety of this intervention cannot be demonstrated with one case nor can the effectiveness. However, our goal is to emphasize and educate readers of these promising results with the hope that this can be the first step toward the required further research in prospective and controlled trials.

## Introduction

Complex regional pain syndrome (CRPS) is a difficult syndrome to effectively treat and manage. Treatment of this disease often requires a multidisciplinary and multimodal approach. CRPS is a disorder affecting both the peripheral and central nervous systems. CRPS is further separated into CRPS-I and CRPS-II depending on the presence and absence of an identified nerve injury. CRPS has classically been described as a process involving sympathetic hyperstimulation. However, recent studies have suggested that while the acute phase may be caused by increased sympathetic tone, the chronic phase (“cold CRPS”) is likely the result of decreased sympathetic efferent outflow and increased sensitivity to catecholamines [1-2]. Self-regulation, self-healing, and health maintenance are key principles of osteopathic medicine. We describe a unique case of the use of counterstrain as a way of relieving symptoms associated with hypertonic myofascial restrictions.

## Case presentation

History

The patient is a 23-year-old female who presented to the office with pain and numbness in her right foot and lower leg that had been gradually intensifying for the past year, following right plantar sesamoidectomy for non-healing fractures. She rated her pain as a 6/10 at rest and a 10/10 if aggravated or paroxysmal. Her pain was worse with prolonged activity and exercise but would also become exacerbated if she remained sitting for a long period of time. Light activity and firm pressure (compression stockings) provided temporary relief. Two months after surgery, she began experiencing pain around her right fibular head and sharp pain at her right first metatarsophalangeal joint. Her pain continued to intensify, developing a cramping-type character as well as increased muscle fatigue and the sensation of intense, localized pressure. She then developed progressive neuropathy in her right foot and lower leg and began experiencing deficits in temperature sensation and vacillations between hot and cold, starting at her popliteal fossa and radiating distally. She also reported paresthesia that would occur either at rest or as a result of light contact with her clothing or her bedsheets that would occasionally wake her from sleep. After nine months of the above symptoms, she began having episodes of severe pain and contracture in the flexor tendons of the plantar aspect of her right foot, which would also wake her from sleep. She had attempted physical therapy, medication trials, and a cam walking boot without relief of symptoms.

At the time of presentation to the clinic, she was lacking discriminatory sensation between hot and cold, and she reported that her leg had been alternating in color and tactile character throughout the day between red and purple and hot and cold, respectively. Her paresthesia was initially limited to her right great toe, the plantar aspect of her foot, the medial aspect of her ankle, and the posterolateral aspect of her right calf, but it had progressed to a “stocking” distribution from her popliteal fossa radiating distally. She had recently begun experiencing deficits in proprioception of her right leg to such a degree that she was having difficulty operating the gas and brake pedals in her car. She also reported pain, numbness, swelling, and paresthesia that originated in her right gluteal region and radiated down her posterior thigh following the distribution of the sciatic nerve. She complained that her leg “felt like lead” and that her “right foot felt like a brick” along with a marked decreased active range of motion in all planes. She noted several episodes of swelling in her foot and leg, to the point where she had difficulty wearing socks or shoes.

Examination 

On initial examination, the patient's right leg was edematous, discolored, and subjectively cool to the touch when compared to her unaffected leg. She had atrophy of her right calf and right quadriceps, with marked restrictions in all planes of both active and passive range of motion-particularly eversion-of her right foot and ankle. Her gait was imbalanced, favoring the right leg while walking. She indicated that walking caused her symptoms to worsen. Sitting for any length of time caused radicular pain that originated from her gluteal region and radiated down to the incision site on her foot. This pain was reproduced by deep palpation of her piriformis.

Prior treatment 

One month after surgery, she began physical therapy twice a week for four months, with no noted improvement of pain, range of motion, or neuralgia. She rotated between crutches and a walking boot for four months after surgery. In December 2016, the patient received orthotic inserts to assist with walking mechanics. In February 2017, she was prescribed gabapentin (100 mg by mouth, three times a day), which was tapered down and discontinued in April 2017 due to the worsening of the original symptoms.

Following her diagnosis of complex regional pain syndrome, she began using a transcutaneous nerve stimulation (TENS) unit three times a day, resulting in temporary pain relief that lasted for a few hours. She received referrals to a neurologist, a pain management specialist, and a physical therapist who specialized in pain syndromes, all of whom managed her above treatment.

Diagnoses 

Complex regional pain syndrome with secondary piriformis syndrome. The diagnosis of complex regional pain syndrome is supported by the Budapest criteria (Table 1) [3], with the patient reporting hyperesthesia and allodynia, temperature fluctuations and asymmetry, edema, and motor weakness, fulfilling four out of four categories. In the clinic, the above signs were observed, fulfilling the diagnostic criteria.

**Table 1:** Budapest clinical diagnostic criteria for CRPS CRPS: complex regional pain syndrome Source: [3]

Pain	Displayed Symptoms	Displayed Signs
Continuing pain disproportionate to the inciting event	Sensory symptoms corresponding to hyperesthesia and/or allodynia. Vasomotor symptoms corresponding to temperature asymmetry, skin color changes, and/or skin color asymmetry. Symptoms corresponding to edema, sweating changes, and/or sweating asymmetry. Motor/trophic symptoms corresponding to a decreased range of motion, motor dysfunction, and/or trophic changes.	Sensory symptoms of hyperalgesia, allodynia (to light touch, deep somatic pressure, and/or joint movement). Vasomotor symptoms corresponding to temperature asymmetry, skin color changes, and/or asymmetry symptoms corresponding to edema, sweating changes, and/or sweating asymmetry. Motor/trophic symptoms corresponding to a decreased range of motion, motor dysfunction, and/or trophic changes.
	Requires at least one symptom in three out of the four categories listed above.	Requires at least one sign in two or more of the categories listed.

The diagnosis of piriformis syndrome was made based on observed dermatomal neuritis symptoms, pain at the ipsilateral sacroiliac joint, and a positive Lasègue’s test [4]. The patient also had a tender point in the center of the piriformis muscle belly that reproduced her symptoms, consistent with the clinical diagnosis [4].

Selected treatment 

This patient was treated with a combination of standard and novel osteopathic manipulative treatment (OMT). Although poorly understood, it is postulated that a hypersympathetic tone and autonomic imbalance play a role in the pathophysiology of complex regional pain syndrome [5]. As such, the first treatment session began with sympathetic inhibition via rib raising, followed by parasympathetic stimulation via sub-occipital release [6]. Once the patient’s autonomic tone was established, tissue textures were assessed from the incision site on her medial right metatarsophalangeal joint ascending to the popliteal fossa. This approach was used in each subsequent treatment session.

Tender points, as described by Jones [7], were identified during initial palpation throughout the affected limb, particularly in the fibularis muscles and in the flexor muscles in the plantar aspect of the foot. These tender points were treated in the usual fashion (counterstrain): light pressure over the tender point to monitor tissue changes while the affected tissues are relaxed around the tender point until a position of maximal comfort, as reported by the patient, is achieved. The patient is held in this position for 90 seconds, after which time he/she is slowly and passively returned to neutral [7]. The previously identified piriformis tender point was treated using traditional counterstrain with the patient prone, the affected leg abducted approximately 70 degrees, with 15 degrees of hip flexion, knee flexion, and the thigh externally rotated. This position produced a 100% reduction of reported pain and was thus held for 90 seconds. This provided an immediate and lasting resolution of the associated symptoms: difficulty sitting for prolonged periods of time, paresthesia, and gluteal pain.

For the treatment of this patient’s complex regional pain syndrome symptoms, a novel combination of continued deep pressure and/or aggravation coupled with counterstrain-style manipulation was used, relying on patient feedback until the initial pain and discomfort of the pressure were completely absent. Rather than identify a position of maximal comfort and hold that position for 90 seconds, minor adjustments were continuously made, including the addition of compression or distraction of the hip, while pressure over the tender point was gently and steadily increased. This technique elicited twitch reflex responses in the targeted tissues, and the treatment of each point was considered complete with the resolution of these twitch responses. Treatment of various tender points in the gastrocnemius, soleus, fibularis longus and brevis, flexor hallucis longus, the intrinsic muscles of the foot, and the plantar fascia was completed using this technique once a week for six sessions, with improvement in reported symptoms at the end of each session.

The novel treatment approach involved treating the allodynia directly. There is no documentation of counterstrain being used to directly treat allodynia or paresthesia, in complex regional pain syndrome patients or otherwise, although it has been used to successfully treat tender points, reduce reported pain, and improve active range of motion in a complex regional pain syndrome patient [8]. Similar to palpating and treating musculoskeletal tender points, focal areas of extreme hypersensitivity were located by using stimuli identified by the patient. For example, clothing such as a pant leg or a sock would elicit painful, radiating paresthesias. It is important to note that these hypersensitive areas did not represent any standard dermatomal distribution, and they were severely deficient in discriminatory touch.

Treatment of these points began in the second treatment session and followed the same approach as counterstrain with continued pressure as outlined above. A point of maximum hypersensitivity was located using an irritating stimulus, and stimulation of that point continued with simultaneous manipulation of the tissues around the point until the painful sensation was reduced by at least 80%, with the treatment goal being a reduction by 100%. This approach elicited the same twitch reflex response as the musculoskeletal points, except these “points” were purely neurological. After cessation of twitch reflex responses was established, each point was reevaluated using the original inciting stimulus, and reduction of hypersensitivity was noted. By the end of the third treatment session, there was an improvement in the patient’s edema, discoloration, and temperature discrimination, as well as improved proprioception. After completing six treatment sessions, the patient’s temperature discrimination was restored completely, save a small area of numbness over the incision site, she had near-complete restoration of her proprioception and felt that she could comfortably drive again and was tolerating physical activity for prolonged periods of time (light jogging for >45 minutes).

## Discussion

In order to elucidate a possible mechanism by which this treatment approach elicited its results, the pathophysiology of complex regional pain syndrome must first be discussed. Given the complex and multifactorial nature of the disease, the following will serve as a survey of the current literature in the field.

Complex regional pain syndrome is a disorder of both the peripheral and central nervous system, including peripheral and central sensitization, local inflammation, changes in autonomic tone (primarily sympathetic hypersensitivity and catecholaminergic receptor up-regulation), changes in somatosensory representation in the brain, and psychophysiological factors [9]. It is further separated into CRPS-I and CRPS-II, differentiated by the absence or presence of an identified nerve injury, respectively [10]. While the research regarding the pathophysiology of complex regional pain syndrome is ongoing, there are several proposed mechanisms that can be used to explain the results observed with this patient. Most of the clinical hallmarks of complex regional pain syndrome can be explained, in part, by autonomic dysregulation, including local edema, bluish skin tone due to excessive vasoconstriction, and regional sweating of the affected area. An increased firing rate of nociceptors in post-ischemic injury animal models was demonstrated when dosed with norepinephrine, which suggests a sympatho-nociceptive nerve coupling [11]. This mechanism is further supported by the expression of adrenergic receptors on nociceptive fibers following trauma to those nerve fibers, which allows for the direct sympathetic stimulation of those nociceptors [12-13]. Although complex regional pain syndrome has historically been assumed to be the result of sympathetic hyperstimulation, recent studies have suggested that while the acute phase (“warm CRPS”) may be caused by increased sympathetic tone, the chronic phase (“cold CRPS”) is likely the result of a combination of decreased sympathetic efferent outflow and increased sensitivity to catecholamines [1-2].

Sensory deprivation and dissociation is another clinical manifestation of complex regional pain syndrome and was evident in this patient at presentation. Several studies have identified possible mechanisms by which the symptoms of complex regional pain syndrome manifest, though large-scale trials are still lacking [14-16]. It is postulated that following the initial trauma from the nerve block and the surgery itself, postoperative inflammation and the resultant decreased circulation allowed for the accumulation of nociceptive neurotransmitters, which likely contributed to this patient’s regional allodynia and hyperesthesia [9].

One of the osteopathic principles is that the body is capable of self-regulation, self-healing, and health maintenance. The osteopathic techniques employed as part of the treatment regimen with this patient were aimed at improving symptoms by treating hypertonic myofascial restrictions. While the mechanisms by which these effects were achieved are not well-understood, this author has several theories. First, by treating the myofascial tissues, blood flow, perfusion, and lymphatic flow would improve. This would allow the removal of metabolic waste products and nociceptive neurotransmitters, replenishing other blood products to the hypoxic tissues. This would have an immediate effect on localized pain and edema, both of which were observed after each treatment. In addition to improving local tissue symptoms, range of motion was greatly improved.

Another osteopathic principle is the belief that when the body is in homeostasis, it is capable of self-healing and maintenance of health. By improving the range of motion, the natural muscular pump that drives venous and lymphatic flow was restored, which allowed for the progressive resolution of her symptoms over the course of several treatments. Tense myofascial tissues not only contribute to local hypoxia but may also directly entrap cutaneous nerves. It is possible that by irritating entrapped nerves and then manipulating the surrounding tissues, as was done with this technique, the myofibroblasts within the nerve sheath were allowed to depolarize and relax, in turn relaxing the nerve sheath and embedded axons. This would then allow for axoplasmic flow to return and any mechanoreceptor-mediated nerve firing to cease.

Osteopathic manipulative medicine has been utilized successfully to treat chronic pain syndromes, and this case report outlines yet another example. However, this is an area of research that is in its infancy and requires further investigation, so we may more fully understand the mechanisms by which clinical results are achieved. Such understanding will allow for improved manipulation techniques and overall improvement of treatment algorithms for chronic pain syndrome patients.

## Conclusions

Counterstrain should be considered a reasonable option to assist in the treatment of complex regional pain syndrome. This new treatment approach does not require extensive training or experience with osteopathic manipulative treatment, nor does it take much time to administer. Thus, it could be easily learned and utilized by many standard practitioners for patients with complex regional pain syndrome. In addition, given its low intensity and passive approach, it more likely to be tolerated well by patients. Understanding the challenging nature of treating complex regional pain syndrome, this report aims to be helpful in adding to the general fund of knowledge regarding this condition and the possible treatments. We are in full understanding that the safety of this intervention cannot be demonstrated with one case, nor can the effectiveness. However, our goal is to emphasize and educate readers of these promising results with the hope that this can be the first step toward required further research in prospective and controlled trials.
